# Human-Centered Design Approaches in Digital Mental Health Interventions: Exploratory Mapping Review

**DOI:** 10.2196/35591

**Published:** 2022-06-07

**Authors:** Stéphane Vial, Sana Boudhraâ, Mathieu Dumont

**Affiliations:** 1 Centre de Recherche de l'Institut Universitaire en Santé Mentale de Montréal École de Design Université du Québec à Montréal Montréal, QC Canada; 2 Département D'ergothérapie Université du Québec à Trois-Rivières Drummondville, QC Canada

**Keywords:** design, human-centered design, user experience, mental health, digital mental health

## Abstract

**Background:**

Digital mental health interventions have a great potential to alleviate mental illness and increase access to care. However, these technologies face significant challenges, especially in terms of user engagement and adoption. It has been suggested that this issue stems from a lack of user perspective in the development process; accordingly, several human-centered design approaches have been developed over the years to consider this important aspect. Yet, few human-centered design approaches to digital solutions exist in the field of mental health, and rarely are end users involved in their development.

**Objective:**

The main objective of this literature review is to understand how human-centered design is considered in e-mental health intervention research.

**Methods:**

An exploratory mapping review was conducted of mental health journals with the explicit scope of covering e-mental health technology. The human-centered design approaches reported and the core elements of design activity (ie, object, context, design process, and actors involved) were examined among the eligible studies.

**Results:**

A total of 30 studies met the inclusion criteria, of which 22 mentioned using human-centered design approaches or specific design methods in the development of an e-mental health solution. Reported approaches were classified as participatory design (11/27, 41%), codesign (6/27, 22%), user-centered design (5/27, 19%), or a specific design method (5/27, 19%). Just over half (15/27, 56%) of the approaches mentioned were supported by references. End users were involved in each study to some extent but not necessarily in designing. About 27% (8/30) of all the included studies explicitly mentioned the presence of designers on their team.

**Conclusions:**

Our results show that some attempts have indeed been made to integrate human-centered design approaches into digital mental health technology development. However, these attempts rely very little on designers and design research. Researchers from other domains and technology developers would be wise to learn the underpinnings of human-centered design methods before selecting one over another. Inviting designers for assistance when implementing a particular approach would also be beneficial. To further motivate interest in and use of human-centered design principles in the world of e-mental health, we make nine suggestions for better reporting of human-centered design approaches in future research.

## Introduction

### Background

E-mental health research is expanding around the world [1]. This area of mental health research and intervention relies on digital technologies to deliver complementary care, support, and information [2]. Over the past ten years, digital mental health interventions have appeared at an unprecedented rate, largely in the form of mobile apps, social media, chatbots, and virtual reality [3]. Since the beginning of the COVID-19 pandemic, the digital health world has expanded at an unprecedented rate [4], and its potential to improve access to care has never been greater [5]. However, some important challenges remain in the field. Several issues raised in recent years have not been resolved: there is some distrust in the field that is not served by the lack of empirical validation of its benefit [6-9]; it raises privacy and data security concerns [9,10]; it presents commercial issues (eg, financial interest, user access, advertising) [11,12]; and the solutions often lack usability and show low user engagement [13,14].

### The Problem of Adoption

The promise of digital technology still far outweighs the reality of its use. This is particularly evident in the field of digital mental health, in which designs must survive successive waves of adoption: phases of preuse, first use, and sustained use [15]. A study of 93 mobile mental health apps showed that the overall user retention is very low, with a 15-day retention rate of 3.9% and a 30-day retention rate of 3.3% [16]. Another study with 77 participants in two randomized controlled trials demonstrated how difficult it is to motivate people to begin using an e-mental health solution [17]. Any digital health trial will see a significant proportion of users drop out or cease using the app before completion. Eysenbach [18] calls this phenomenon “the law of attrition.” Data on the health app market are scarce but do converge on two findings: the majority of health apps are downloaded fewer than 5,000 times, and 46% of apps have less than one monthly active user [19]. While usage is only one indicator of engagement [20], these statistics are consistent with adoption issues commonly reported among users, such as a lack of awareness of the app or lack of time and motivation to use one [21,22]. This is concerning because the use of these apps may not be associated with a significant decrease in mental disorders if they are not used for the intended period of time [14]. The average cost of developing a mobile health app is US $425,000 [19]—a cost-benefit ratio too high and unsustainable in the long run if we do not change how they are developed.

### Lack of Attention to User Perspectives During the Design Process

Research has shown that most people are willing to adopt and use some form of new technology in the interest of improving their mental health [23]. So why the low utilization rates? Given the already significant barriers to adoption that users face (eg, privacy concerns, commercial issues), we seek to underscore the importance of user-centric design approaches for the development of e-mental health solutions, of which a solid notion is lacking in the digital mental health design sphere [14]. Based on the existing literature [13], we hypothesize that the lack of adoption of digital mental health solutions could be largely due to a lack of attention to user perspective in the design of these technologies, or at the very least, a lack of understanding of design approaches that accommodate user perspectives. In the field of mental health, there are very few examples of involving real people with mental disorders in the development and design of mobile apps intended for them [14]. The most common development approaches seem primarily researcher- and expert-driven, top-down in style, and to rely mainly on a bilateral partnership between clinicians and engineers [24]. This is not adapted to the challenges of contemporary digital culture that places the user at the center of these platforms by empowering them [25].

### Design Principles and Human-Centered Design Approaches

In this section, we recall some fundamental principles of design culture and explain how they can help actors better account for the needs of users and integrate their perspective early on in the e-mental health design process.

#### Designers and Engineers

According to Cumulus [26], an international association of art and design education and research, a designer is someone who has acquired professional design expertise at a “design school.” For instance, Jony Ive, former chief design officer at Apple, is an industrial designer who graduated from the Northumbria School of Design in the United Kingdom. Although engineers might be considered designers (according to the broad sense of the word “design” in English), design and engineering are two separate fields that correspond to two different professions, methods, and cultures. Nevertheless, they share some similarities; for instance, both interaction designers and software engineers follow an iterative process [27].

However, design must not be confused with engineering design, as differences in the way engineers and designers tackle the design of a technology are well documented [28]. In the initial prototyping phases, engineers seek to define specific goals to be achieved and, following a linear way of thinking, focus on technical functioning. Designers, on the other hand, use prototypes to creatively explore the design space for novel possibilities [29]. In health care, designers tend to focus attention on unmet needs and ways to improve care and are sensitive to how care is received through user-centered practices [30]. In this paper, we use the term “human-centered design” to distinguish the field of design from engineering, and when we say “design,” we mean human-centered design.

####  Core Elements of Design Activity: Actor, Object, Context, Process

It is generally admitted in the field of design studies that the core elements of design activity are the following: (1) a design problem and its coevolving design solution are its *objects*; (2) the environment in which design activity takes place is the design *context*; (3) the structure and dynamics of design activity are the *process*; and (4) a designer (person, team, organization) is an *actor* [31-33]. To be clear, let us consider the example of Temstem, an app developed in the Netherlands based on language games intended to help people experiencing psychosis distract themselves from voices they hear in their minds [34]. Temstem was co-designed by a group of designers, psychotherapists, and people living with psychosis—all of whom constitute *actors* (4). In collaboration with Parnassia Group, a private nonprofit mental health institution, a group of industrial design students from the Delft University of Technology spent a day in the life of people with psychosis. This led to a solution fully designed by the Amsterdam-based Reframing Studio design firm—all of which constitute the *context* (2). Design students were asked to come up with a product that would promote the recovery of psychosis—which constitutes the “problem” aspect of the *object*—and this product turned into an app called Temstem (“tame voices” in Dutch) to help people cope with “hearing voices”—constituting the “solution” aspect of the *object* (1). The main methods used to imagine and build this solution were co-design, user experience design, interaction design, game design, and ethnographic approach — the core components of the *process* (3).

#### Typical Process of a Design Activity

**Figure 1:**
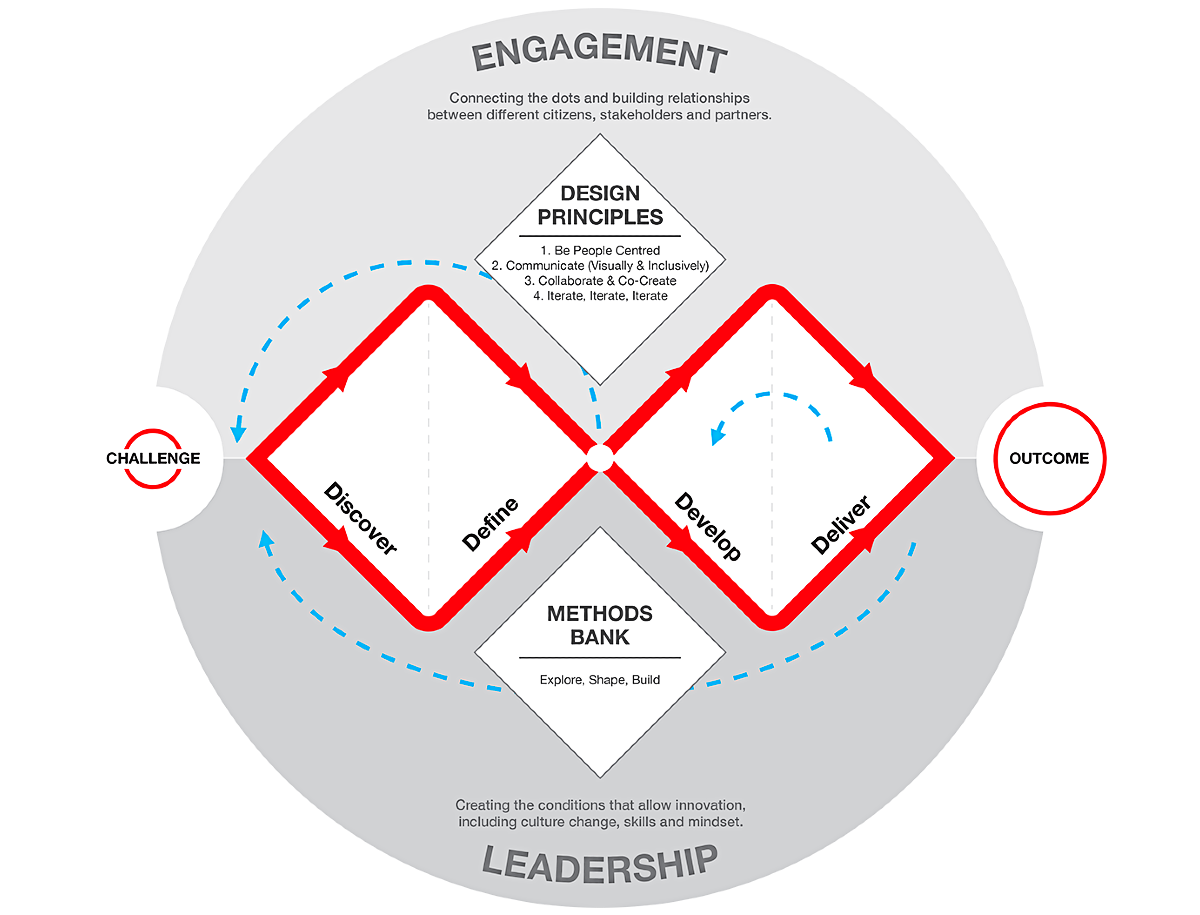
Framework for Innovation (used with permission from Design Council 2019).

Design research is a relatively young field that appeared in the 1960s and is represented today by the International Association of Societies of Design Research [35]. Since its inception, this field has focused on the study of the design process [31]. The design process has also been the subject of research outside of academia to help the profession structure its methods. In 2005, the Design Council in the United Kingdom published the first version of its Double Diamond model, which was updated in 2019 and renamed the Framework for Innovation [36]. This internationally recognized model proposes a schematic representation of the typical process of any human-centered design activity (Figure 1).

The framework comprises 4 steps: (1) *discover* (ie, gather information, understand the problem, make sense of them, and broaden the possibilities); (2) *define* (ie, narrow down the possible paths and define the main challenge); (3) *develop* (ie, give different answers to the clearly defined problem and push further the most promising solution, mostly by prototyping); and (4) *deliver* (ie, test and refine different versions of the solution at different scales). Each step is associated with specific and relevant methods. For instance, the design methods for step 1 include user diaries and quantitative surveys, whereas the design methods for step 2 employ techniques such as focus groups and customer journey mapping. The value of the Double Diamond is that it captures what all human-centered design approaches have in common from the perspective of the *process*.

#### Human-Centered Design Approaches

There are several human-centered design approaches that allow end users to significantly and positively impact the design of technologies. The most used are user-centered design, user experience design, design thinking, participatory design, and co-design. These design methods stem from industry practice rather than academia and are very advanced there. They are used by design agencies, communications agencies, and large technological companies. These methods are historically derived from the disciplines of industrial and graphic design and from the evolution of the latter in contact with digital technologies [37]. Supported by the works of influential authors in design studies, we present how the five differ from each other and for what purpose each is generally used.

User-centered design, also called human-centered design, was defined in the late 1980s by Don Norman in his book *The Design of Everyday Things* [38]. It is used to design products that are readily usable and immediately understandable thanks to the observance of certain design principles, such as the salience of affordances (ie, when the user understands what to do just by looking). Enriched by the work of J Nielsen on web usability [39] and JJ Garrett on user experience [40], user-centered design has become the standard for best practices in web design. Garrett defines it as “the practice of creating engaging and effective user experiences,” which involves considering the user at every stage of product development [40]. User-centered design is the fundamental basis of many current practices in modern industrial design, UX design, and interaction design. It is used to gain the best possible knowledge of end users’ needs and desires and to transform this knowledge into the best possible design of a product through usability testing. User-centered design should be used to validate the product’s utility, efficiency, and desirability.

User experience design, also known as UX design, is about optimizing the experience that arises from interacting with a product, service, or technology [41]. User experience is defined as “the experience that the product creates for the people who use it in the real world,” meaning not its internal workings but “the way it works externally, where a person comes into contact with it” [40]. In the case of an app, it is the cognitive and emotional experience that the user has in front of the screen. In the field of digital technologies, the expression “UX design” has now largely replaced “user-centered design.” UX design should be used to create meaningful interfaces and engaging interactive experiences: it will make it more useful, more attractive, and more engaging for the end user.

Design thinking as a human-centered method has been widely theorized, practiced, and popularized by the IDEO design agency and its founders. It can be defined as “a creative method of innovation, based on design-like culture and designer-like methods, whose main focus is on the needs of its end users,” and it has three dimensions: the desirability, feasibility, and viability of the future product or service [42]. There are important similarities between user-centered design and design thinking approaches—two terms that appeared around the same time—mainly the central place given to empathy and listening to the user's needs. Design thinking is recognized worldwide for its ability to foster the emergence of user-centered innovative solutions through cocreation, including in the field of health care [43]. It is generally used to implement transformations inside an organization, stimulate creativity within a team, or devise new solutions in a specific sector. Design thinking should be used to drive innovation in an organization or a team to make them more creative and empathetic with end users and to build better products and technologies.

Participatory design was first defined in Norway and Sweden in the 1970s and 1980s by Kristen Nygaard and Pelle Ehn, respectively. Its original objective was to involve users in every stage of the design and development process of a complex computer system by using low-tech mediation techniques that are easy to handle by nonexperts (colored notes hung on the wall, cardboard mock-ups, decks of design cards) [44]. Participatory design is used to involve users in design activities such as ideation or prototyping. The approach is often implemented partially or even incorrectly, typically reduced to inviting end users to participate during the beginning of the process for research needs or at the end for usability testing [45,46]. Gathering feedback from users via usability testing is not a form of participatory design since users are not involved in the actual designing act of the design process.

Co-design is often used as a synonym for “participatory design.” However, the term actually refers to a specific form of participatory design that is much closer to cocreation. Co-design refers to the creativity of designers and people not trained in design combined during the design development process. It is “collective creativity as it is applied across the whole span of a design process” [47]. It is a truly participatory approach in which the user is engaged from the start as an equal partner and has been widely recognized as a lever for social innovation [48].

Participatory design and co-design are generally used to better consider the needs and desires of users in the design of a product and to make the design process less top-down and more democratic. These two approaches aid in developing an idea early that is in line with users' realities and to engage them in the product before it even exists. Co-design, in particular, should be used when the team is faced with a complex problem and seeks to improve and evolve its initial idea, provided that it accepts that the participants can transform this idea in a meaningful way.

All these human-centered design approaches must become more familiar, better understood, and more widely implemented in the field of mental health in general and e-mental health in particular.

### Objective and Research Questions

The main objective of this literature review was to understand how human-centered design is considered in e-mental health intervention research. The following research questions were considered:

1. Which human-centered design approaches are reported in the development of e-mental health interventions?
2. How are these approaches used in light of the generally accepted core elements of a design activity?
3. How are designers involved in the process and what roles are they given?

Through our efforts, we seek to open the discussion on the place of human-centered design methods in e-mental health research.

## Methods

### Study Design

To answer the 3 questions, an exploratory mapping review was conducted by researchers from the fields of design and mental health. The aim of mapping reviews is to map out and categorize existing literature on a particular topic to identify gaps in knowledge or opportunities for further research [49]. It focuses less on findings and more on activities related to the findings, such as the quantity and quality of the literature [49,50]. To streamline the process and identify a relevant sample of articles for this interdisciplinary exploratory review, a search was conducted among journals in mental health whose explicit scope covers technology. The following journals were identified: *JMIR Mental Health*, *Frontiers in Psychiatry*, *Internet Interventions*, and the *Journal of Technology in Behavioral Science.* Articles published between 2015 and 2020 were examined using the search terms “design” and “design*” to narrow down the results.

Given the interdisciplinary nature of this work, extensive discussions were conducted among the coauthors to agree on a common understanding of the concept of design. The following inclusion criteria were defined: articles reporting original research on the development of a digital technology in mental health and those addressing the concept of design (at least one explicit use of the term design) in connection with at least one core element of a design activity.

The use of the term “design” in research (eg, “study design”) or in its common sense was excluded.

### Study Selection

The third author (MD) screened all titles and abstracts for potential articles. Then, the second (SB) and third (MD) authors independently assessed the full text of the articles for eligibility. There was an initial level of agreement of 80.7% (42/52) between the two authors (SB and MD), which is usually considered acceptable in the literature [51]. When there was discrepancy, the first author (SV) made the final decision.

### Data Extraction and Analysis

For each article selected, data regarding the design approaches and the four core elements of a design activity (ie, the object, the context, the actor, and the process) were extracted. This included the type of solution created, the design approaches reported, the setting in which the project took place, and the type of actors involved throughout the design process. The design process was examined according to the steps defined in the UK Design Council’s framework for innovation [36]. The design methods reported in the articles were used to define the steps addressed in the development of the digital solutions. The analysis process was conducted jointly by the second (SB) and third (MD) authors.

## Results

### Overview

Of the 1035 articles initially found, 51 full-text articles were assessed for eligibility. Of these, 30 studies met the inclusion criteria. The articles came from *JMIR Mental Health* (22/30, 73%), *Frontiers in Psychiatry* (4/30, 13%), *Internet Interventions* (2/20, 7%), and the *Journal of Technology in Behavioral Science* (2/30, 7%). Multimedia Appendix 1 presents the characteristics of the included studies, indicated from left to right: the specific research domain (eg, depression and anxiety, psychosis, well-being, etc), the synthesized naming of the adopted approach, and the 4 core elements of a design activity (object, context, process, and actors). Process is presented according to the 4 steps in the Double Diamond (coding each actor type with a number across the steps). Finally, we reported whether the study indicated that the process was iterative or not.

### Design Approaches

To develop the digital solutions, 22 studies mentioned using human-centered design approaches or specific design methods. Various design approaches were reported, and there were many variations in the names given to these approaches. After several rounds of discussions between all authors, different approaches were classified under the 3 common names used in design studies, as listed in Table 1: participatory design (11/27, 41%), co-design (6/27, 22%), and user-centered design (5/27, 19%). Under the term “participatory design,” generally named as such in the studies, we considered alternative names such as “user involvement” [52]. Under the term “user-centered design,” we included other names like “person-based approach” or “person-centered approach.” Other studies reported specific design methods (5/27, 19%) that did not correspond to these 3 common names. Those methods are not common in the design studies field, except for the UK Design Council’s Double Diamond method. Among the studies included in this review, 8 (27%) did not refer to any human-centered design approach, so they are not listed in Table 1 [53-60]. Five studies reported more than one approach, mixing 2 common approaches or 1 common approach with 1 specific design method, and these studies are demarcated with a superscript in Table 1. Only 16 studies provided a definition of the reported approach(es), either by referring to other studies (15/16, 94%) or by offering their own definition (1/16, 6%). This means that about half (14/30, 47%) of the studies did not cite or provide a definition for their chosen approach, include references, or mention the theoretical underpinnings of the design approach. Although it was the second most reported approach, co-design was never defined in the 6 studies that mentioned it.

**Table 1:** Classification of the reported approaches.

Reported approaches		Authors	Reported definition
**Participatory design approach**			
	Participatory design	Peters et al [61]	Yes
	Participatory design (explore, approximate, refine framework)	Buitenweg et al [62]	Yes
	Participatory design thinking and methods	Terp et al [63]	Yes
	Participatory design (using research and development cycle)	Ospina-Pinillos et al [64]	Yes
	User-involvement processes	Buus et al [52]	Yes
	Participatory design process	Cheng et al [65]	Yes
	Participatory design approach	Reupert et al [66]	Yes
	Participatory design methods	Gulliver et al [67]	Not reported
	Participatory design	Werner-Seidler et al [68]	Not reported
	Participatory design process	Peck et al [69]	Not reported
	Participatory design	Povey et al [70]^a^	Not reported
**Co-design approach**			
	Co-design approach	Yoo et al [71]^a^	Not reported
	Iterative co-design process	Christie et al [72]	Not reported
	Co-design process	Povey et al [70]^a^	Not reported
	Co-design	Torous et al [73]	Not reported
	Co-design	Bevan Jones et al [74]^a^	Not reported
	Human-centered co-design	Hetrick et al [75]^a^	Not reported
**User-centered approach**			
	User-centered approach	Honary et al [76]	Yes
	(Aligned with) person-based approach	Abraham et al [77]	Yes
	User-centered approach	Stawarz et al [78]	Yes
	Person-based/person-centered approach; user-centered approach	Bevan Jones et al [74]^a^	Yes
	User-centered design research	Hardy et al [79]^a^	Not reported
**Specific design methods**			
	Design research framework	Terlouw et al [80]	Yes
	Iterative approach informed by the ADDIE^b^ framework	Khan et al [81]	Yes
	UK Design Council’s Double Diamond method	Hardy et al [79]^a^	Not reported
	Agile design development/design studio methodology	Hetrick et al [75]^a^	Yes
	Needs-affordances analysis framework	Yoo et al [71]^a^	Yes

^a^Authors who reported using more than one approach.

^b^ADDIE: Analyze, Design, Develop, Implement, and Evaluate.

### Core Elements of the Design Activity

#### Object (Solution)

The digital technologies developed were mobile apps (15/30, 50%), web platforms (10/30, 33%), desktop apps (2/30, 7%), virtual reality (1/30, 3%), a serious game (1/30, 3%), and a digital comic creator (1/30, 3%). The solutions were most often used for applications related to anxiety and depression (8/30, 27%), well-being (5/30, 17%), access to and quality of care (5/30, 17%), and psychosis (4/30, 13%).

#### Context

Most (23/30, 77%) design activities were conducted exclusively in academic environments. Some studies (5/30, 17%) mentioned a collaboration with a private company. Two studies reported either a collaboration with community organizations (1/30, 3%) or public mental health services (1/30, 3%). The collaborative work took place within projects using participatory design (3/11, 27%), co-design (3/6, 50%), or no identifiable approach (1/8, 13%).

#### Process

About two-thirds of the projects adopted an iterative process (21/30, 70%). Most of the studies (27/30, 90%) described methods including at least 3 out of the 4 steps of the Double Diamond framework. As one might expect, the studies that covered fewer steps were those not reporting any identified design approach. For 10 studies (indicated by superscript 'a' in Multimedia Appendix 1), the *discover* and *define* steps were not clearly differentiated. The most often missing step was *deliver*, which was planned but not carried out in 8 of the studies (at the time these papers were published).

#### Actors

All 30 studies mentioned that end users were involved at some point in the process, but not necessarily in the act of designing (Table 2). Designers were explicitly mentioned in only 8 studies, whereas software development companies were mentioned in 14. We know from experience that software companies include few UX designers on their teams in proportion to the number of software engineers (eg, even in a small team of 5 software engineers, we can find at best 1 UX designer). However, there were no details about this in the 14 studies. A few studies reported involving other actors such as experts (consultants, health professionals; 8/30, 27%) and various stakeholders (eg, advocates, philanthropists; 1/30, 3%).

**Table 2:** Distribution of the explicitly mentioned actors.

Actors	Explicitly mentioned in the 30 studies, n (%)
Designers	8 (27)
Software development company	14 (47)
Experts (including health professionals, consultants)	8 (27)
End users	30 (100)
Community of interest	1 (3)

### Involvement of Designers

Although the 30 studies selected addressed the concept of design and reported a variety of human-centered design approaches, very few explicitly mentioned the presence of designers on their teams. Regardless of the step of the process, only 8 studies mentioned designers, representing about 27% of all included studies. For the 22 studies that did not mention them (74%), we do not know whether it is because no designer was involved or because the presence of designers was not considered important enough to be reported.

Looking at the few studies mentioning designers in their teams (8/30, 27%), it is interesting to note that some designers were explicitly present for all steps but mostly just the first three: *discover* (4/8, 50%), *define* (3/8, 38%), and *develop* (7/8, 88%). Only 1 was explicitly present for *deliver.* It is also interesting to note that 3 of the 4 studies that included designers in the *discover* step also included them in the *define* and *develop* steps, reflecting their ongoing involvement in the process. These 3 studies represented a small proportion of the studies that reported using participatory design and co-design. Overall, designers were clearly more involved in the *develop* step (7/30, 23%) but much less involved here than software development companies (Table 3). The latter were exclusively present in this step (14/30, 47%). End users were the most present participants at each step of the design process (Table 3).

**Table 3:** Distribution of the explicitly mentioned actors according to the 4 steps of the Double Diamond (discover: n=27; define: n=27; develop: n=30; deliver: n=17).

Actors	Steps			
	Discover, n (%)	Define, n (%)	Develop, n (%)	Deliver, n (%)
Designers	4 (15)	3 (11)	7 (23)	1 (6)
Software development company	0 (0)	0 (0)	14 (47)	0 (0)
Experts (including health professionals, consultants)	3 (11)	5 (19)	5 (17)	1 (6)
End users	24 (89)	26 (96)	21 (70)	15 (88)
Community of interest	0 (0)	0 (0)	1 (3)	1 (6)

## Discussion

### Principal Results

In this initial exploratory research study, we investigated how design is considered in e-mental health research. Our results show that there have been attempts to integrate human-centered design methods into the development of e-mental health solutions, but they are still rare and rely very little on designers or design research. Most reported design approaches such as user-centered design, participatory design, and co-design are well known and documented in the design research literature, but most of the included studies did not rely on them. Almost half of the included studies did not bring or report any existing definition of the design approach they used. Moreover, it was not possible to link the use of an approach to its influence on the main core elements (steps conducted through the process or actors involved) and vice versa. The impact of each chosen approach on the whole process is not clear, nor is the reason behind the selection of a particular approach. This indicates that there is a lack of shareable knowledge on how design approaches are understood, and by extension, applied in the mental health field. This suggests that human-centered design methods are not fully integrated in e-mental health and that reported design approaches are still primarily used from the outside without a deep understanding of the design culture that is needed to fully leverage their power.

### Comparison With Earlier Work

There has been very little research conducted on human-centered design methods in e-mental health and on how to guide the design of e-mental interventions. Thabrew et al [82] highlight the importance of active collaboration using co-design jointly between researchers, designers, developers, and users to develop more engaging and useful interventions. The results from this literature review show that such collaboration among all these stakeholders remains limited throughout the design process. While most design approaches reported were consistent with human-centered methods stemming from the design discipline, the choice and combination of the approaches varied greatly across studies. Orlowski et al [83] claim that the e-mental health development process must prioritize empathy and understanding over innovation, as proposed in participatory design and design thinking approaches. Torous et al [14] highlight the poor usability of mental health apps and the lack of user-centric design. Aryana et al [84] attempt to identify the key principles of the design process relevant to mobile mental health. Among the 6 principles identified, they mention “high quality user experience,” which is closely related to user-centered design, and an “empathic design process,” which is closely related to participatory design and co-design, and conclude that there are few examples of the implementation of several of these design principles in real-world products. This was also the case for the identified research projects. Bakker et al [85] note that design principles that have led to the huge success of many physical health and social networking apps have not been utilized in the mental health apps field. These findings are all consistent with our study and show that human-centered design methods are largely underutilized and neglected when their impact could be very important, especially on user engagement.

### Limitations

This exploratory review offers significant insights into how design is considered in e-mental health. We consider it to show a fairly representative sample of the type of design-related research currently being conducted on the development of digital technology in mental health. We do not think that additional studies would significantly change our main conclusions. However, this study does not meet the criteria for a systematic review and has a few limitations. First, when analyzing the core elements of design activity, we could only rely on the information reported in the articles, which was fairly heterogeneous. We had to conduct several rounds of interdisciplinary discussions among ourselves (the authors) to ensure its best interpretation. Second, to analyze the design process described in each study, we chose the Double Diamond framework, which is a global reference, but other frameworks could also be used and might yield additional results. Third, in all the studies selected, it was difficult to understand how end users influenced the design, especially in participatory approaches. User involvement can be informative, consultative, or fully collaborative [86]. Orlowski et al [46] have already concluded that it is difficult to track ongoing user participation and clearly determine the contribution of participatory design to the effectiveness of designed interventions.

### Research Implications

#### Good Design Comes Before Effective Science

Health technologies are useless if they are not used, even if they are validated by science. We urge health researchers and technology developers in e-mental health to consider human-centered design methods not as the form-giving step of a technology development process but as a comprehensive approach integrated at an early stage in close relation to the research strategy and vision. Researchers and technology developers in e-mental health should consider systematically hiring interaction designers, user interface designers, user experience designers, and service designers in their teams to fully implement the human-centered design approach they need and then increase user engagement and technology acceptance. They should also include co-design workshops with end users conducted by trained designers from the beginning to the end of the development process. Design comes before science, which means that in the realm of apps, good design is a prerequisite for effective science.

#### Suggested Recommendations for Better Reporting of Human-Centered Design Approaches

This study suggests that researchers in e-mental health may not understand or value design principles enough to clearly describe them in their manuscripts. Without claiming to define a publication standard for reporting the design process and the outcomes of that process, we suggest 9 recommendations to be considered to further motivate interest in and adoption of design principles and human-centered design approaches (Textbox 1).

**Recommendations to motivate interest in and adoption of design principles and human-centered design approaches.**

**Name and definition of human-centered design approaches**

1. Explicitly state which human-centered design approach was used.

2. Provide a definition or, at least, a reference for each human-centered approach.

3. Explain why a human-centered design approach is chosen (for which purpose).

**Implementation of the core elements of a human-centered design activity**

4. Describe each of the 4 core elements: object, context, process, and actors.

5. Clearly define the steps and the methods used in the design process. If necessary, use a framework such as the Framework for Innovation (Figure 1).

6. Explain when and to what extent actors were involved in the design process.

**Involvement of designers**

7. Indicate how many designers (not engineers or software developers) are involved.

8. Specify what design profession they practice (UX designer, interaction designer, service designer, design researcher, etc).

9. Indicate if the designers contribute on their own behalf or if they are employed by software development companies.

### Future Work

Bridging the gap between design and e-mental health is our next research agenda. We are currently developing a health intervention research framework called Design For e-Mental Health [87]. This framework refers to the broad range of human-centered design creative strategies that define the structure, function, and form of a digital mental health with a high quality of experience in terms of user experience, scientific validity, privacy, and viability.

## Appendix

Multimedia Appendix 1 Classification of the included studies.
