# Supplementary material for: Human-Centered Design Approaches in Digital Mental Health Interventions: Exploratory Mapping Review
Source: JMIR Ment Health. 2022 Jun 7;9(6):e35591. doi: 10.2196/35591 (PMC9214621; doi:10.2196/35591)
Supplement: Multimedia Appendix 1 [file mental_v9i6e35591_app1.docx]

**Multimedia Appendix 1.** Classification of the included studies.

Legend: 1 researchers; 2 designers; 3 design and software development company; 4 end users (e.g., patients, practitioners, caregivers); 5 experts (eg, practitioners, scholars); 6 Various stakeholders

| Authors | Domain | Approach | Solution | Context | Design process | | | | Iterative |
| --- | --- | --- | --- | --- | --- | --- | --- | --- | --- |
|  |  |  |  |  | Discover | Define | Develop | Deliver |  |
|  |  |  |  |  |  |  |  |  |  |
| Abraham et al [77] | Depression and anxiety | User-centered | Desktop app | Academic | (1, 4, 5)^a^ | (1, 4, 5)^a^ | (1, 2) | N/A^b^ | ✓ |
| Honary et al [76] | Emotional support | User-centered | Web platform | Academic | (1, 4)^a^ | (1, 4)^a^ | (1, 3, 4) | (1, 4) |  |
| Stawarz et al [78] | Depression | User-centered | Web platform | Academic | (1, 4)^a^ | (1, 4)^a^ | (1, 4) | (1, 4) | ✓ |
| Buitenweg et al [62] | Quality of life | Participatory design | Mobile app | Academic | (1, 4) | (1, 4) | (1, 3) | (1, 4) | ✓ |
| Buus et al [52] | Suicide | Participatory design | Mobile app | Academic | (1, 4)^a^ | (1, 4)^a^ | (1, 3, 4) | FW^C^ | ✓ |
| Cheng et al [65] | Well-being | Participatory design | Mobile app | Academic | (1, 4) | (1) | (1, 3, 4) | (1, 4) | ✓ |
| Gulliver et al [67] | Access and quality of care | Participatory design | Web platform | Academic | (1, 4) | (1, 4) | (1, 4) | N/A | ✓ |
| Ospina-Pinillos et al [64] | Access and quality of care | Participatory design | Web platform | Academic | (1, 4) | (1, 4) | (1, 4, 5) | FW | ✓ |
| Peck et al [69] | Psychosis | Participatory design | Web platform | Collaboration (mental health services) | (1, 4) | (1, 4) | (1, 4) | FW |  |
| Peters et al [61] | Well-being | Participatory design | Mobile app | Collaboration (community organization) | (1, 4) | (1, 4) | (1) | (1) |  |
| Reupert et al [66] | Well-being | Participatory design | Web platform | Academic | (1, 2, 4) | (1, 2, 4) | (1, 2, 4) | (1, 4) | ✓ |
| Terp et al [63] | Psychosis | Participatory design | Mobile app | Collaboration (software company) | (1, 4) ^a^ PW^d^ | (1, 4)^a^ | (1, 3, 4) | (1, 4) | ✓ |
| Werner-Seidler et al [68] | Sleep | Participatory design | Mobile app | Academic | (1, 2, 4)^a^ | (1, 2, 4)* | (1, 2, 4) | FW | ✓ |
| Christie et al [72] | Depression and anxiety | Codesign | Mobile app | Collaboration (software company) | (1, 4)^a^ PW | (1, 4)^a^ PW | (1, 3, 4, 5) | FW | ✓ |
| Torous et al [73] | Access and quality of care | Codesign | Mobile app and platform | Collaboration (software company) | (1, 4) | (1, 4) | (1, 2, 3, 4, 6) | (1, 6) | ✓ |
| Khan et al [81] | Depression | Specific method | Mobile app | Academic | (1, 2, 5, 4 )* | (1, 5, 4) ^a^ | (1, 5, 3, 4) | (1, 5, 4) | ✓ |
| Terlouw et al [80] | Autism Spectrum Disorder | Specific method | Digital Comic Creator | Academic | PW | (1, 4, 5) PW | (1, 2, 5) | (1, 4) | ✓ |
| Bevan Jones et al [74] | Depression | User-centered; codesign | Web platform | Academic | (1, 4) | (1, 4) | (1, 4, 3) | FW | ✓ |
| Hardy et al [79] | Psychosis | User-centered; specified method | Serious game | Academic | (1, 4) | (1, 4) | (1, 2, 3, 4) | (1, 4) | ✓ |
| Hetrick et al [75] | Depression | Codesign; specific method | Mobile app | Collaboration (software company) | (1, 2, 4) | (1, 2, 4) | (1, 2, 4) | N/A | ✓ |
| Povey et al [70] | Access and quality of care | Participatory design; Codesign | Mobile app | Academic | (1, 4) ^a^ | (1, 4) ^a^ | (1, 3, 4, 5) | FW | ✓ |
| Yoo et al [71] | Access and quality of care | Codesign; specific method | Desktop app | Academic | (1=4) | (1=4) | (1=4) | N/A | ✓ |
| Baron et al [53] | Sleep | Not reported | Mobile app | Academic | N/A | N/A | (1) | (1, 4) |  |
| Doherty et al [54] | Well-being | Not reported | Mobile app | Academic | (1, 4) | (1, 4) | (1, 4) | N/A | ✓ |
| Hoffman et al [55] | Anxiety | Not reported | Web platform | Academic | (1,4, 5)^a^ | (1,4, 5) ^a^ | (1, 3) | (1, 4) |  |
| Ijaz et al [56] | Cognitive impairment (memory) | Not reported | Virtual reality | Academic | N/A | N/A | (1, 4) | FW |  |
| Lim et al [57] | Loneliness | Not reported | Mobile app | Academic | (1, 4)^a^ | (1, 4)^a^ | (1, 4) | (1, 4) | ✓ |
| Rickard et al [58] | Well-being | Not reported | Mobile app | Collaboration (software company) | N/A | N/A | (1, 3) | (1, 4, 3) |  |
| Thomas et al [59] | Psychosis | Not reported | Web platform | Academic | (1, 4) | (1, 4) | (1) | (1, 4) |  |
| Tiburcio et al [60] | Depression and substance abuse | Not reported | Web platform | Academic | (1) | (1, 4, 5) | (1, 3) | (1, 4) |  |

^a^Indicates that Discover and Define steps were not clearly differentiated by the authors in those studies.

^b^N/A: not applicable.

^C^FW: future work.

^d^PW: previous work.
